# Diabetes mellitus, periapical inflammation and endodontic treatment outcome

**DOI:** 10.4317/medoral.17452

**Published:** 2011-12-06

**Authors:** Juan J. Segura-Egea, Lizett Castellanos-Cosano, Guillermo Machuca, Jose López-López, Jenifer Martín-González, Eugenio Velasco-Ortega, Benito Sánchez-Domínguez, Francisco J. López-Frías

**Affiliations:** 1 Department of Endodontics, School of Dentistry, University of Sevilla, C/ Avicena s/n, 41009 Sevilla, Spain; 2 Department of Stomatology, School of Dentistry, University of Sevilla, C/ Avicena s/n, 41009 Sevilla, Spain; 3 Department of Odonto-stomatology, School of Dentistry, University of Barcelona, Campus d Bellvitge, C/ Feixa Llarga s/n, 08907 L’Hospitalet, Barcelona, Spain

## Abstract

The possible connection between chronic oral inflammatory processes, such as apical periodontitis and periodontal disease (PD), and systemic health is one of the most interesting aspects faced by the medical and dental scientific community. Chronic apical periodontitis shares important characteristics with PD: 1) both are chronic infections of the oral cavity, 2) the Gram-negative anaerobic microbiota found in both diseases is comparable, and 3) in both infectious processes increased local levels of inflammatory mediators may have an impact on systemic levels. One of the systemic disorders linked to PD is diabetes mellitus (DM); is therefore plausible to assume that chronic apical periodontitis and endodontic treatment are also associated with DM. The status of knowledge regarding the relationship between DM and endodontics is reviewed. Upon review, we conclude that there are data in the literature that associate DM with a higher prevalence of periapical lesions, greater size of the osteolityc lesions, greater likelihood of asymptomatic infections and worse prognosis for root filled teeth. The results of some studies suggest that periapical disease may contribute to diabetic metabolic dyscontrol.

** Key words:** Apical periodontitis, diabetes mellitus, endodontics, root canal treatment.

## Introduction

Apical periodontitis (AP) is an acute or chronic inflammatory lesion around the apex of a tooth caused by bacterial infection of the pulp canal system. Periradicular lesions consecutive to AP result from a periapical inflammatory response provoked by polymicrobial irritants from root canals. AP is a remarkably prevalent problem (1). In Europe, the prevalence of AP rises 61%, increasing with patients age (2). When apical periodontitis has occurred treatment is aimed at restoring the periradicular tissues to health: this is usually carried out by root canal treatment, occasionally in combination with surgical endodontics. In Europe, the prevalence of endodontic treatment is estimated around 41% (2).

Although the periradicular infectious process produces a variety of local tissue responses with the likely purpose to confine and limit the spreading of the infectious elements, AP may not exclusively be a local phenomenon. In its non-balanced acute stage, spreading of the infection and the inflammatory process to nearby tissue compartments is possible and may bring about severe, but fortunately rare, fatal inflammatory conditions. Moreover, considering the increasing awareness of a potential relationship between persistent, inflammatory disorders of the oral cavity and disease conditions in other organs of the body, acute and chronic manifestations of AP may also be implicated (3).

The possible connection between chronic oral inflammatory processes of infectious origin, i.e. chronic apical periodontitis and periodontal disease (PD), and systemic health is one of the most interesting aspects faced by the medical and dental scientific community. In the two last decades several epidemiological studies have investigated the association between systemic health and PD. Thus, PD has been associated to diabetes mellitus (DM) (4,5), coronary heart disease (CHD) (6) and acute myocardial infarction (AMI) (7), preterm-low birth weight (8), respiratory diseases (9) and osteoporosis in post-menopause women (10). The evidence of the association between PD and systemic diseases has increased the attention at the diagnosis and treatment of PD, improving, consequently, the patient’s oral and systemic health (11).

Although some differences are evident between chronic periodontal and periapical inflammatory processes, both show three important resemblances: 1) both are chronic infections of the oral cavity, 2) both are polymicrobial infections sharing a common microbiota with predominance of Gram-negative anaerobic bacteria (12), and 3) elevated cytokine levels may be released syste-mically from acute and chronic manifestations of both disease processes (e.g., increased concentrations of inflammatory mediators have been detected both in the gingival crevicular fluid of subjects with periodontal disease and in the periapical tissues of endodontically involved teeth) (13). 

Likewise, it can be assumed that AP is linked with the same systemic disorders associated to PD (3). Consequently, numerous investigations have been conducted to study the relationship between AP and coronary heart disease (13,14), hypertension (15-17), and smoking (18). Moreover, several studies have analyzed the possible association between AP and DM, a clinically and genetically heterogeneous group of disorders affecting the metabolism of carbohydrates, lipids and proteins, in which hyperglycaemia is a main feature. In this paper, the current status of knowledge regarding the relationship between AP and DM is reviewed.

## Diabetes Mellitus

Diabetes mellitus (DM) is a group of complex multisystem metabolic disorders due to a deficiency in insulin secretion caused by pancreatic β-cell dysfunction and/or insulin resistance in liver and muscle. Diabetes affects more than 9% of the adult population and has a dramatic impact on the health care system through high morbidity and mortality among affected individuals (19).

Type 1 diabetes results from cellular-mediated autoimmune destruction of pancreatic β-cells, which usually leads to total loss of insulin secretion; in contrast, type 2 diabetes is caused by resistance to insulin combined with a failure to produce enough additio-nal insulin to compensate for the resistance. Type 2 diabetes is commonly linked to obesity, which contributes to insulin resistance through elevation of circulating levels of free fatty acids derived from the adipocytes; these free fatty acids inhibit glucose uptake, glycogen synthesis and glycolysis. In many obese individuals, insulin resistance is compensated by increased insulin production. However, in one-third of obese individuals, β-cell mass is reduced by a marked increase in β-cell apoptosis, which results in inadequate production of insulin (20).

## Association between diabetes mellitus, periapical status and the outcome of root canal treatment

DM affects many functions of the immune system and is associated with delayed healing and compromised immune responses (21). DM-induced changes in immune cell function produce an inflammatory immune cell phenotype (up-regulation of pro-inflammatory cytokines from monocytes / polymorphonuclear leukocytes and down-regulation of growth factors from macrophages). This predisposes to chronic inflammation, progressive tissue breakdown, and diminished tissue repair capacity (22). Evidence has consistently indicated that diabetes is a risk factor for increased severity of gingivitis and periodontitis (23). So, it is plausible to hypothesize that DM predisposes to oral infection and could also act as a risk factor for AP, increasing the rate of root canal treatment failure. Several studies have tried to answer this hypothesis.

 Animal studies

The relationship between endodontic infections and DM has been investigated in animal models. Kohsaka et al. (24) studied histologically and histometrically the changes in pulpal and periapical tissues after pulpal exposure in streptozotocin-induced diabetic rats. In experimental rats, inflammation in the apical periodontal ligament and root resorption and alveolar bone resorption were more severe than that in control rats. Fouad et al. (25) induced periapical lesions in first molars of female nonobese diabetic (NOD) mice and measured periapical lesion size histomorphometrically, finding a more severe response in diabetic mice compared with controls. Iwama et al. (26) evaluated the effects of type 2 diabetes on the development of periradicular lesions after exposure of the pulp in the left mandibular first molar through the occlusal surface in Goto-Kakizaki (GK) rats with spontaneous non-insulin-dependent diabetes mellitus and Wistar rats (controls). Four weeks after pulp exposure, histologic analysis showed that alveolar bone resorption was most severe and the periradicular lesions were largest in diabetic rats given a sucrose solution, suggesting that the metabolic conditions produced by type 2 diabetes enhance the development of periradicular lesions in rats. Recently, Garber et al. (27) have studied the effect of hyperglycaemia on pulpal healing in exposed rat pulps capped with mineral trioxide aggregate. Two groups of 11 rats received injections of saline (control group) or streptozotocin to induce hyperglycaemia (DM group). The pulps of the maxillary first molars of all rats were exposed and capped. Intact teeth and teeth with exposed pulps without restorations served as positive and negative controls, respectively. Dentin bridge formation was inhibited in diabetic rats (p = 0.029) along with more inflammation in these pulps (p = 0.005). There was an inverse association between dentin bridge formation and inflammatory cell infiltration (p = 0.001). Based on these results, the authors conclude that it appears that hyperglycemia adversely affects pulpal healing in rats.

 Human studies

The literature on the pathogenesis, progression, and healing of AP in diabetic patients is scarce. Bender et al. (28) reported that, in cases of poorly controlled DM, periapical radiolucencies tend to develop during treatment but, if DM is under therapeutic control, periapical lesions heal as readily as in non-diabetics. Cheraskin & Ringsdorf (29) monitored radiographically the healing of peri-radicular lesions following root canal treatment in twelve patients with low plasma glucose and thirteen patients with high glucose. After thirty weeks, the periradicular radiolucencies in the low glucose groups were reduced by an average of 74 percent compared with a reduction of only 48 percent for the high glucose group. Bender & Bender (30) found a high rate of asymptomatic tooth infections in diabetics exhibiting poor glycaemia levels of an unclear cause. Falk et al. (31) conducted a clinical and radiographic investigation showing a greater prevalence of periapical lesions in type 1 diabetics. They observed that women with long diabetes duration exhibited more root-filled teeth with periapical lesions than women with short diabetes duration and women without diabetes. Long duration diabetics exhibited teeth with more periapical lesions than the other groups. Ueta et al. (32) studied the prevalence of DM in odontogenic infections reporting that patients with DM had a disproportionately high percentage of clinically severe pulpal or periodontal infections (24% of all cases), but had a much lower percentage of moderate infections (2.3%), concluding that DM was a predisposing condition for endodontic infections. Fouad et al. (25) described the association of *Porphyromonas gingivalis* and *Porphyromonas endodontalis* isolated in samples from root canals with necrotic pulp and a history of diabetes mellitus (OR > 2), but the sample was too small to establish any definitive association.

Fouad & Burleson (33) investigated endodontic diagnostic and treatment outcome data in patients with and without diabetes. A multivariate analysis showed that patients with diabetes have increased periodontal disease in root-filled teeth and have a reduced likelihood of success of root canal treatment in cases with preoperative periradicular lesions. Britto et al. (34) investigate the prevalence of radiographic periradicular radiolucencies in root-filled teeth and untreated teeth in patients with and without diabetes. Results showed that men with type 2 diabetes who had root canal treatments were more likely to have residual lesions. In a retrospective cohort study, Segura-Egea et al. (35) determined radiographycally the prevalence of AP in patients with and without type 2 diabetes mellitus. Results showed that apical periodontitis in at least one tooth was found in 81.3% of diabetic patients and in 58% of control subjects (p = 0.036; OR = 3.2, 95% C.I. = 1.1 - 9.4). Amongst diabetic patients 7% of the teeth had AP, whereas in the control subjects 4% of teeth were affected (p = 0.007; OR = 1.8, 95% C.I. = 1.2 - 2.8). Mindiola et al. (15) carried out an epidemiological study of a regional population of Native Americans identifying factors affecting the retention of root-filled teeth and to determine frequencies of endodontic care. The results suggested that diabetes contributes to decreased retention of root-filled teeth. Doyle et al. (36), in a retrospective study, evaluated whether diabetes was associated with the outcome of patients undergoing non-surgical root canal treatment, finding a borderline significantly association (p = 0.063). Wang et al. (17) analyzed the long-term prognosis of teeth receiving non-surgical root canal treatment (NSRCT) in patients with DM to elucidate the impact of DM on the risk of tooth extraction after NSRCT. Results showed that DM was a significant risk factor for tooth extraction after NSRCT (p < 0001; OR = 1.8). A recent prospective epidemiologic study, using self-reported history of root canal therapy, concluded that diabetes was more prevalent among patients with CHD with 24 or fewer teeth reporting never having had endodontic treatment (14).

Since diabetes is the third most prevalent condition in medically compromised patients seeking dental treatment (37), dentists should be aware of the possible relationship between endodontic infections and diabetes and take it into account in the attention to diabetic patients.

## Possible effect of periapical infection on diabetes mellitus

It has been stated that PD can have a significant impact on the metabolic state in diabetes. The presence of periodontitis increases the risk of worsening of glycemic control over time (11). It has been proposed that PD could initiate or propagate insulin resistance in a similar manner to that of obesity, by enhancing activation of the overall systemic immune response initiated by cytokines (5). Several biologically plausible mechanisms could be proposed to explain the interactions between diabetes and PD. Type 2 diabetes is a manifestation of the host’s inflammatory response, because an ongoing cytokine-induced acute-phase response (a low-grade inflammation that occurs through activation of the innate immune system) is closely involved in the pathogenesis of this disease (20). Likewise, the mechanisms of the host-mediated response in PD involve activation of the broad axis of innate immunity, specifically by up-regulation of proinflammatory cytokines from monocytes and polymorphonuclear leukocytes. Thus, chronic gram-negative periodontal infections may induce or perpetuate an elevated chronic systemic inflammatory status, contributing to increased insulin resistance and poor glycemic control (11).

As it has been exposed previously, there are important similitudes between PD and AP. So, it could be hypothesized that chronic periapical inflammatory processes can also contribute to the pathogenesis of DM, being a risk factor for worsening glycaemia control among diabetic patients. Some investigations have analyzed this topic. Bender et al. (28) reported that inflammatory periapical reactions are greater in diabetic states, and the increased local inflammation causes an intensification of diabetes with a rise in blood glucose, placing the patient in an uncontrolled diabetic state. This often requires an increase in insulin dosage or therapeutic adjustment. Removal of the inflammatory state usually creates a need for a lesser amount of insulin for diabetic control. Thus, it becomes axiomatic to remove all infections including those of the dental pulps. Schulze et al. (38) described the effects of an acute focal dental inflammation and subsequent root canal treatment on the required insulin dosage of a 70-year-old man who had moderately controlled diabetes. This case report shows a highly relevant correlation between insulin resistance and a local dental inflammation of endodontic origin. 

The mechanisms of the effect of chronic periapical infections on diabetic patients must be similar to that existing

**Figure 1:**
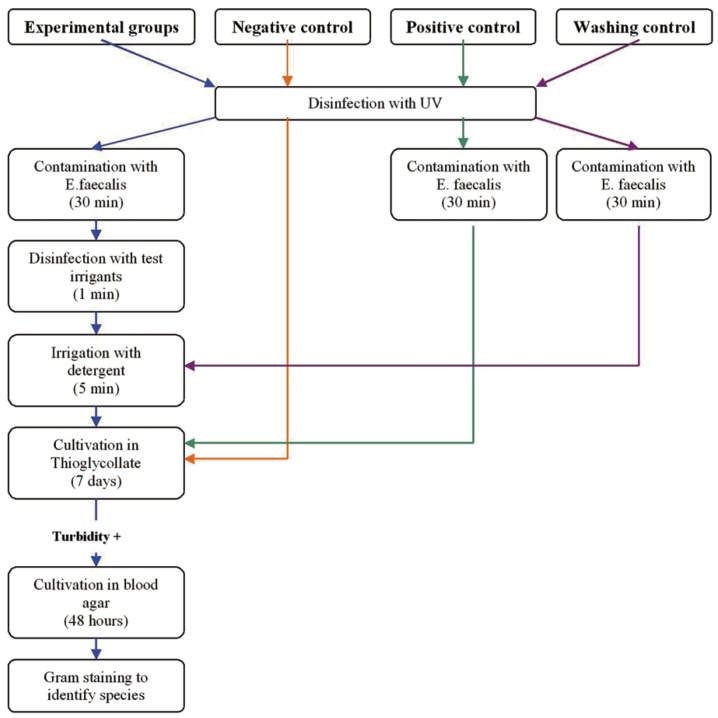
Interaction between endodontic infection and diabetes mellitus. LPS: lipopolysaccharide; NF-kβ: nuclear factor kappaβ; CAP: chronic apical periodontitis.

between PD and DM (Fig. 1) (20). Chronic inflammation through the action of inflammatory mediators is mainly associated with the development of insulin resistance, which is influenced by genetically modified environmental factors, including decreased physical activity, poor nutrition, obesity and infection (39). Chronic apical periodontitis involves activation of the broad axis of innate immunity. The lipopolysaccharide (LPS) from anaerobic gram-negative bacteria causing apical periodontitis activates intracellular pathways (nuclear factor kappa B, NF-kβ) on macrophages and neutrophils, upregulating pro-inflammatory cytokines such as IL-1β, IL-6, IL-8, tumour necrosis factor alpha (TNF-α) and prostaglandin E2 (PGE2). These locally produced cytokines move into the systemic circulation (36), where they interact with the free fatty acids and advanced products of glycosylation (AGEs), characteristic of type 2 DM. The activation of these inflammatory pathways in immune cells (monocytes or macrophages), endothelium cells, adipocytes, hepatocytes and muscle cells could promote an increase in the overall insulin resistance, altering the metabolic control in patients with both type 2 diabetes and chronic apical periodontitis.

The results of studies conducted so far are not conclusive, but suggest an association between DM an AP. There is evidence from the literature associating DM with higher prevalence of AP, greater size of the periapical osteolytic lesions, greater likelihood of asymptomatic periapical infections, and delay / arrest of periapical repair. The prognosis for root filled teeth is worse in diabetics, showing a higher rate of root canal treatment failure with increased prevalence of persistent chronic apical periodontitis. The results of some studies suggest that chronic periapical disease may contribute to diabetic metabolic dyscontrol. Prospective epidemiological studies are needed to deepen the relationship between DM and periapical inflammation.
